# Current clinical understanding and effectiveness of portopulmonary hypertension treatment

**DOI:** 10.3389/fmed.2023.1142836

**Published:** 2023-04-04

**Authors:** Yuichi Tamura, Yudai Tamura, Yu Taniguchi, Masanori Atsukawa

**Affiliations:** ^1^Pulmonary Hypertension Center, International University of Health and Welfare Mita Hospital, Tokyo, Japan; ^2^Department of Cardiology, International University of Health and Welfare School of Medicine, Narita, Japan; ^3^Division of Cardiovascular Medicine, Department of Internal Medicine, Kobe University Graduate School of Medicine, Kobe, Japan; ^4^Division of Gastroenterology and Hepatology, Nippon Medical School, Tokyo, Japan

**Keywords:** portopulmonary hypertension, treatment, endothelin receptor antagonist, liver transplantation, pulmonary arterial hypertension, screening

## Abstract

Portopulmonary hypertension (PoPH) is a rare subtype of Group 1 pulmonary arterial hypertension (PAH) with a poor prognosis. According to the most up-to-date definition, PoPH is characterized by a mean pulmonary arterial pressure (PAP) of >20 mmHg at rest, a pulmonary artery wedge pressure of ≤15 mmHg, and a pulmonary vascular resistance (PVR) of >2 Wood units with portal hypertension. Like PAH, PoPH is underpinned by an imbalance in vasoactive substances. Therefore, current guidelines recommend PAH-specific therapies for PoPH treatment; however, descriptions of the actual treatment approaches are inconsistent. Given the small patient population, PoPH is often studied in combination with idiopathic PAH; however, recent evidence suggests important differences between PoPH and idiopathic PAH in terms of hemodynamic parameters, treatment approaches, survival, socioeconomic status, and healthcare utilization. Therefore, large, multi-center registry studies are needed to examine PoPH in isolation while obtaining statistically meaningful results. PoPH has conventionally been excluded from clinical drug trials because of concerns over hepatotoxicity. Nevertheless, newer-generation endothelin receptor antagonists have shown great promise in the treatment of PoPH, reducing PVR, PAP, and World Health Organization functional class without causing hepatotoxicity. The role of liver transplantation as a treatment option for PoPH has also been controversial; however, recent evidence shows that this procedure may be beneficial in this patient population. In the future, given the shortage of liver donors, predictors of a favorable response to liver transplantation should be determined to select the most eligible patients. Collectively, advances in these three areas could help to standardize PoPH treatment in the clinic.

## 1. Introduction

Pulmonary hypertension (PH) is the overarching term used to describe a complex group of conditions that are characterized by loss and obstructive remodeling of the pulmonary vascular bed, leading to an increase in pulmonary vascular resistance (PVR) and pulmonary arterial pressure (PAP). These changes in PVR and PAP cause strain on the right side of the heart, and if this persists for a prolonged period, right-sided heart failure and functional decline can occur (1, 2).

In the World Health Organization (WHO) clinical classification (3), portopulmonary hypertension (PoPH) is positioned as a subtype of Group 1 pulmonary artery hypertension (PAH), and registry data suggest that PoPH accounts for 5–16% of cases of PAH (4–9). PoPH develops in 1.1–6.3% of patients with portal hypertension (10–13), and although most cases in this patient population are related to cirrhosis, non-cirrhotic causes of portal hypertension leading to PoPH have also been noted, including portal vein thrombosis, granulomatous disease, autoimmune diseases, drug reactions, infections (such as hepatitis C), and congenital abnormalities (such as congenital portosystemic shunt) (14–17). The prevalence of PoPH in liver transplant patients is 5–10% (18, 19), and among those with advanced liver disease, such as patients undergoing liver transplantation, women have a higher risk of developing PoPH than men; however, liver disease severity does not appear to be directly related to the risk of PoPH (11).

In terms of the diagnosis of portal hypertension, patients can be diagnosed by the presence of clinical signs, such as ascites, varices, or both, as well as splenomegaly, portal vein dilation, portal vein occlusion, collateral vessel formation, and declining platelet counts (20). Imaging studies, such as Doppler ultrasonography, computed tomography, and magnetic resonance imaging, as well as blood tests, are used to diagnose portal hypertension and determine the presence of the abovementioned features (20). Portal venous pressure is a product of the portal blood flow volume and the resistance to flow; however, direct measurement of portal pressure is not routine due to its invasive nature. A less invasive and indirect measure is the hepatic venous pressure gradient (HVPG), which is considered the best surrogate indicator of portal hypertension in patients with cirrhosis (21). In healthy individuals, the HVPG is 2–5 mmHg, while an HVPG of ≥6 mmHg constitutes portal hypertension and an HVPG of ≥10 mmHg constitutes clinically significant portal hypertension (20). The HVPG is calculated by subtracting the wedged hepatic venous pressure from the free hepatic venous pressure, which are determined by fluoroscopy (20).

In terms of the diagnosis of PH, many patients present as outpatients with symptoms, such as dyspnea, fatigue, or syncope. Others come to the attention of the clinician during screening evaluations, and some present acutely as inpatients (22). Once PH is suspected, echocardiography is commonly used to assess the tricuspid regurgitant velocity, pulmonary artery systolic pressure, and right ventricular wall thickness and function, and right heart catheterization may also be performed (23). Other tests may involve a clinical history and examination, complete pulmonary function testing, thoracic computed tomography, chest radiography, and nocturnal plethysmography to evaluate sleep-disordered breathing (23). The previous right heart catheterization criteria for the diagnosis of PoPH were a mean PAP of ≥25 mmHg at rest, a pulmonary artery wedge pressure of ≤15 mmHg, and a PVR of >3 Wood units with portal hypertension (9). However, the latest definition specifies a mean PAP of >20 mmHg (24). Certain et al. (25) also recently proposed a lower cut-off PVR value of 2 Wood units based on its benefit in achieving an early diagnosis. The cut-off value for pulmonary artery wedge pressure remains at ≤15 mmHg (24). For PoPH specifically, serological analysis for markers of liver failure will also be performed (23).

**Figure 1:**
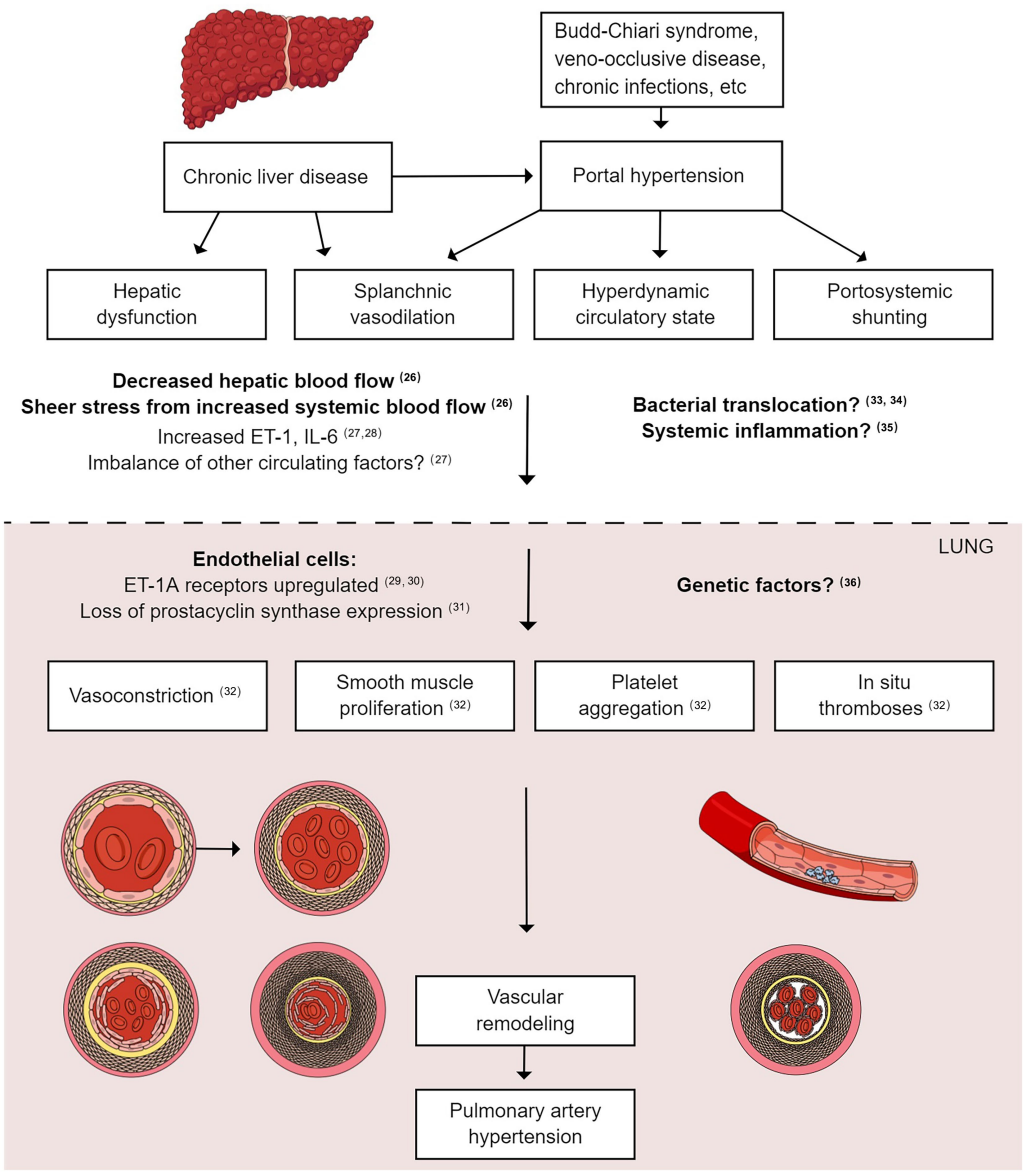
Pathophysiology of portopulmonary hypertension. Liver cirrhosis increases intrahepatic resistance and increases the portal pressure gradient, leading to portal hypertension. The reperfusion/dilation of existing vessels causes portosystemic collateralization. Vasoactive substances bypass the liver and evade hepatic metabolism due to portosystemic shunting, leading to a hyperdynamic state. Endothelin-1 and interleukin-6 are among the circulating substances that reach the pulmonary vasculature, leading to net vasoconstriction and an increase in PVR. Smooth muscle cell proliferation, platelet aggregation, and *in situ* thrombosis also occur, leading to vascular remodeling and PAH. PAH, pulmonary arterial hypertension; PVR, pulmonary vascular resistance.

The pathogenesis of PoPH has been reviewed in detail previously (17) (Figure 1) (26–36). Briefly, cirrhosis causes an increase in intrahepatic resistance and an increased portal pressure gradient, which leads to portosystemic collateralization through the reperfusion/dilation of existing vessels and the generation of new vessels (37). At the molecular level, portosystemic shunting causes blood containing vasoactive substances to bypass the liver, thus evading hepatic metabolism. This reduction in peripheral vascular resistance, combined with indirect vasodilation *via* intestinal vasoactive substances that bypass the liver and reach the systemic circulation, culminates in a hyperdynamic state (37). Endothelin-1 and interleukin-6 are among the substances that are thought to increase in PoPH (38, 39), and this imbalance in vasoactive substances (such as endothelin-1) and pro-inflammatory cytokines (such as interleukin-6) in the pulmonary vasculature leads to net vasoconstriction and an increase in PVR. Thus, one commonly accepted pathogenic mechanism of PoPH is an imbalance in vasoactive substances in the pulmonary circulation in patients with cirrhosis (40, 41). Like PoPH, PAH also results in an imbalance in vasoactive substances and circulating factors; therefore, European Society of Cardiology and European Respiratory Society guidelines (42) recommend that PoPH treatment should follow that of PAH.

The 5-year survival rate of untreated patients with PoPH is as low as 14.2% (24); however, despite its poor prognosis, the rarity of this condition means that descriptions of the clinical features and treatment approaches for PoPH are scarce and inconsistent (43). A number of studies examining the treatment approaches to PoPH have been published in recent years (Table 1), many of which have not yet been reviewed. In this review, we aim to provide an up-to-date analysis of recent literature to establish the current clinical understanding and effectiveness of PoPH treatment. We will briefly discuss the reasons for the limited knowledge of PoPH; consider the controversies around studying PoPH in combination with idiopathic PAH; discuss treatment trends, including the potential of newer-generation endothelin receptor antagonists; and consider evidence for the usefulness of liver transplantation in PoPH patients. We will also provide our expert opinion on how this knowledge could be used to design future clinical trials to deepen the understanding of PoPH and standardize treatment in the clinic.

**Table 1:** Summary of recently published studies.

Publication	Study design	Population	Intervention/ Exposure	Comparator	Outcomes
Sitbon et al. (44)	Multi-center, randomized controlled phase 4 trial	PoPH and Child–Pugh class A/B (*n* = 85)	Intervention: Macitentan (10 mg) for a 12 week double-blind period, followed by a 12 week open-label period	Placebo for a 12 week double-blind period, followed by a 12 week open-label period	35% reduction in PVR with macitentan; 84% (macitentan) and 79% (placebo) of patients experienced adverse events; 21% (macitentan) and 14% (placebo) experienced serious adverse events; most frequent adverse event was edema (macitentan: 26% vs. placebo: 5%); no hepatic safety concerns
Preston et al. (45)	Multi-center, open-label, phase 3 trial	PoPH and Child–Pugh class A/B (*n* = 31)	Intervention: Ambrisentan for 24 weeks, followed by long-term extension for 24–28 weeks	Without ambrisentan (and treatment-naïve) at baseline	Significant reduction in PVR (7.1 ± 5 vs. 3.8 ± 1.8 Wood units); no change in 6-MWD; RAP, mPAP, and CI improved; PCWP unchanged; significant improvement in WHO functional class; most common drug-related adverse events were edema (38.7%) and headache (22.5%)
Savale et al. (46)	Prospective cohort study	PoPH (*n* = 637)	Exposure: Monotherapy with PDE-5i, ERA, or a prostacyclin analog with or without liver transplantation	Dual therapy or triple therapy with or without liver transplantation	Patients treated with dual therapy had a significantly greater median change in PVR than those treated with monotherapy; in the overall cohort, survival from PoPH was better in those who underwent liver transplantation than in those who did not (92, 83, and 81% at 1, 3, and 5 years, respectively, vs. 84, 69, and 51%); in survivors of liver transplantation, PAH therapy was simplified from combination to monotherapy in 16% and discontinued in 22%
DuBrock et al. (47)	Cross-sectional study	PoPH (*n* = 57) vs. I/H-PAH (*n* = 344)	Exposure: PoPH	I/H-PAH	Patients with PoPH had similar WHO functional class, 6-MWD, and mPAP and a higher CI than patients with I/H-PAH; fewer PoPH patients received combination therapy (46.4% vs. 62.2%) and ERAs (28.6% vs. 55.1%) at enrollment, but treatment was similar between PoPH and I-PAH at follow-up; patients with PoPH had more ED visits and hospitalizations in the 6 months preceding enrollment
Salvador et al. (48)	Registry study	PoPH (*n* = 237) vs. I/H-PAH (*n* = 678)	Exposure: PoPH	I/H-PAH	Patients with PoPH were predominantly male, older, had a better WHO functional class, and had better hemodynamics; heart failure biomarkers were worse in PoPH patients; age- and sex-adjusted 5 year survival rate from diagnosis was 49.3% for PoPH and 68.7% for I/H-PAH; PAH- and liver-related causes accounted for 30.2 and 24.7% of deaths, respectively, in PoPH patients; PoPH patients less frequently received PAH-specific therapy, but first-line treatment with PAH-specific therapy was associated with better survival; 3.4% of patients underwent liver transplantation
Takahashi et al. (16)	Retrospective cohort study	PoPH (*n* = 82) vs. I/H-PAH (*n* = 1,112)	Exposure: PoPH	I/H-PAH	Patients with PoPH had higher CO and CI values and lower PVR; fewer PoPH patients received combination therapy; overall and disease-specific survival were similar between PoPH and I/H-PAH
Atsukawa et al. (49)	Retrospective database study	PoPH (*n* = 386)	N/A	N/A	Treatment preferences in PoPH patients: loop diuretics (70.2%), pulmonary vasodilator monotherapy or combination therapy (37.0%), prostacyclin (prostaglandin I_2_) monotherapy (8.8%), ERA + nitric oxide combination therapy (7.0%)
Tamura et al. (9)	Retrospective database study	PoPH (*n* = 62)	Intervention: Combination therapy (≥2 PAH-specific drugs)	Monotherapy	Mean PAP, PVR, and CI were significantly improved with combination therapy

CI, cardiac index; CO, cardiac output; ED, emergency department; ERA, endothelin receptor antagonist; I/H-PAH, idiopathic/heritable pulmonary arterial hypertension; mPAP, mean pulmonary arterial pressure; PAH, pulmonary arterial hypertension; PCWP, pulmonary capillary wedge pressure; PDE-5i, phosphodiesterase type-5 inhibitor; PoPH, portopulmonary hypertension; PVR, pulmonary vascular resistance; RAP, right atrial pressure; WHO, World Health Organization; 6-MWD, 6 min walk distance.

## 2. Reasons for the limited knowledge of PoPH

Specific knowledge of PoPH is limited, and it is relatively understudied compared with other subtypes of Group 1 PAH. There are several possible reasons for this. First, PoPH has conventionally been excluded from drug trials because of concerns about hepatotoxicity (50). For example, in a previous study, bosentan (an endothelin receptor antagonist) led to an elevation in transaminases in approximately 10% of patients with Group 1 PAH without previous liver disease (51). Second, the low incidence of PoPH means that patients with this disease are often studied in combination with patients having idiopathic PAH to ensure sufficient sample sizes. Idiopathic PAH is defined as PAH of unknown cause under the WHO functional classification (3). Thus, the disease etiologies are highly variable, and the mechanisms that underpin disease development in specific patients may vary substantially. Multiple causes of PoPH have also been reported (14–17); however, case numbers are small, which further adds to the complexity of studying this condition in large enough numbers to obtain statistically meaningful results. Therefore, grouping PoPH patients with idiopathic PAH patients to study the effects of drug treatment means that the observations may not necessarily be an accurate reflection of the PoPH population. Third, PoPH has a lower prevalence than other complications, such as hepatic encephalopathy and ascites, in patients with portal hypertension. Therefore, the diagnosis of PoPH might be less of a priority for hepatologists who must diagnose and treat various complications in patients with liver cirrhosis.

## 3. Review of recent literature

### 3.1. Controversy in studying PoPH collectively with idiopathic PAH

Given the low incidence of PoPH, it is often studied in combination with idiopathic PAH when examining the effects of drug therapy. However, differences have been identified between these two patient populations, which suggests that they should be studied independently wherever possible (Table 1). For example, Takahashi et al. (16) extracted data on patients with PoPH from the National Research Project on Intractable Disease in Japan and compared them with data on patients with idiopathic PAH. Patients with PoPH had a higher cardiac output, higher cardiac index, lower PVR, and better 6 min walk distance than patients with idiopathic PAH. In another recent study, DuBrock et al. (47) studied health disparities and treatment approaches between PoPH and idiopathic PAH as part of the Pulmonary Hypertension Association Registry. Dissimilar to Takahashi et al. (16), the authors found that patients with PoPH had a similar 6 min walk distance to patients with idiopathic PAH, as well as a similar WHO functional class and mean PAP. However, similar to Takahashi et al. (16), they identified a higher cardiac index in patients with PoPH than in patients with idiopathic PAH.

In the study by Takahashi et al. (16), although treatments were similar between patients with PoPH and those with idiopathic PAH, the use of prostaglandin I_2_ and endothelin receptor antagonists was lower, and the use of phosphodiesterase type 5 inhibitors was higher in patients with PoPH than in patients with idiopathic PAH. Similarly, in DuBrock et al.’s study (47), fewer PoPH patients than idiopathic PAH patients underwent treatment with endothelin receptor antagonists, including macitentan (28.6% vs. 55.1%, respectively), at enrollment. Moreover, fewer PoPH patients than idiopathic PAH patients were treated with combination therapy (46.4% vs. 62.2%, respectively) at enrollment. However, treatment was similar between PoPH and idiopathic PAH at follow-up. Interestingly, patients with PoPH had more emergency department visits and hospitalizations in the 6 months before enrollment than patients with idiopathic PAH, which could suggest that the addition of endothelin receptor antagonists at follow-up (50% at follow-up vs. 28.6% at enrollment) was effective in reducing the rate of hospitalizations and emergency department visits. This observation corroborates the findings of recent studies by Sitbon et al. (44) and Preston et al. (45), which also demonstrated the beneficial effects of the endothelin receptor antagonists macitentan and ambrisentan, respectively, in patients with PoPH. Initial phosphodiesterase type 5 inhibitor monotherapy was initiated for most PoPH patients with preserved cardiac output and a lower PVR, and a second pulmonary vasodilator (endothelin receptor antagonist) was added sequentially if the improvement in mean PAP was not sufficient. Prior to the clinical trials on macitentan and ambrisentan, endothelin receptor antagonists were not often used as first-line agents in patients with PoPH because of their hepatotoxicity. Therefore, phosphodiesterase type 5 inhibitors were selected first, followed by endothelin receptor antagonists.

Overall, DuBrock et al. (47) showed that patients with PoPH had a worse socioeconomic status, were less likely to be treated with combination therapy at enrollment, and had increased healthcare utilization than patients with idiopathic PAH. However, the study noted that the sample size was too small to detect racial/ethnic differences and differences in survival between patients with PoPH and those with idiopathic PAH.

Adding to the differences between PoPH patients and idiopathic PAH patients identified by DuBrock et al. (47), the Spanish Registry of PAH (48) showed that patients with PoPH were predominantly male and had a better functional class and better hemodynamics than patients with idiopathic PAH. Similar to DuBrock et al.’s study (47), patients with PoPH were less likely to receive PAH-targeted therapy, which was associated with greater mortality. Moreover, first-line PAH monotherapy was associated with better survival. The Spanish Registry of PAH (48) also identified a significant difference in survival between PoPH and idiopathic PAH, reporting age- and sex-adjusted 5-year survival rates of 49.3 and 68.7%, respectively.

Taken together, this recent evidence illustrates important differences between the PoPH and idiopathic PAH populations, including differences in hemodynamics at diagnosis and differences in the therapeutic response to monotherapy, emphasizing the need for large-scale, multi-center trials to enable the PoPH population to be studied in isolation.

### 3.2. Potential of newer-generation endothelin receptor antagonists and treatment trends in PoPH

Although PoPH has conventionally been studied in combination with idiopathic PAH, Sitbon et al. (44) conducted the first randomized controlled trial of PAH therapy in a specific PoPH patient population. The trial adopted a prospective, multi-center, phase 4 study design, comparing the effects of macitentan with placebo in patients with PoPH without severe hepatic impairment. At baseline, 63.5% of patients were undergoing background PAH therapy. Preston et al. (45) conducted another prospective, multi-center, open-label trial in which patients were treated with ambrisentan for 24 weeks, followed by a long-term extension of 24–28 weeks. However, unlike Sitbon et al.’s study (44), patients were treatment-naïve. Importantly, in the study of Sitbon et al. (44), PVR was reduced by 35% in the macitentan group compared with the placebo group, with no hepatic safety concerns. A similar observation was made in the study of Preston et al. (45), in which ambrisentan was associated with a reduction in PVR.

Despite their effects on reducing PVR without hepatic safety concerns, macitentan (44) and ambrisentan (45) had no effect on 6 min walk distance. Moreover, macitentan (44) had no effect on mean right atrial pressure, while ambrisentan improved right atrial pressure as well as mean PAP and cardiac index. However, pulmonary capillary wedge pressure remained unchanged (45). Macitentan did not reduce WHO functional class (44); however, ambrisentan led to a significant improvement in WHO functional class (45). However, direct and simple comparisons of efficacy between these drugs may not be appropriate because the study design (open-label or double-blind) and sample size differed between these studies. For example, the clinical trial on ambrisentan included treatment-naïve patients, while more than half of the patients (64%) in the clinical trial on macitentan were already undergoing other treatments. This could explain the differences in the results between the two trials.

Given their ability to reduce mean PAP and WHO functional class (ambrisentan) and PVR (macitentan and ambrisentan), which are the defining features of PoPH, the endothelin receptor antagonists macitentan and ambrisentan illustrate great promise as therapeutic options for PoPH without causing hepatotoxicity (44, 45, 52), which is a fundamental reason why patients with PoPH have conventionally been excluded from clinical drug trials. However, it should not be disregarded that despite showing promising effects overall, macitentan and ambrisentan have been associated with adverse side effects, such as hypersensitivity, alveolitis, PAH worsening, anemia, peripheral edema, and headache (44, 45), which should be monitored in future trials.

Two recent studies on the current trends in PoPH therapy have been published in Japan. A recent database study by Atsukawa et al. (49) showed that of 386 Japanese patients with PoPH, the combined proportion of patients treated with pulmonary vasodilator monotherapy or combination therapy was 37.0% within 90 days (less than half of patients). Prostacyclin (prostaglandin I_2_) was used in 8.8% of patients within 90 days, and combination therapy with endothelin receptor antagonists plus nitric oxide was used in 7.05% of patients; thus, the use of vasodilators in patients with PoPH remains low.

The low proportion of patients treated with vasodilator therapy (49) is surprising given the beneficial effects demonstrated with these agents. For example, in the Japan Pulmonary Hypertension Registry, Tamura et al. (9) evaluated current treatment patterns and clinical events, as well as changes in hemodynamic and clinical parameters associated with PAH-specific therapy. The results showed that mean PAP, PVR, and cardiac index were significantly improved in the combination therapy group (defined as treatment with ≥2 PAH-specific drugs administered simultaneously during the follow-up period), although the improvement was not significant in the monotherapy group. There were no significant differences in mortality, PH worsening, PAH-specific drug discontinuation due to side effects, or WHO functional class improvement between the monotherapy and combination therapy groups.

Taken together, this new evidence suggests that although vasodilator therapy, and endothelin receptor antagonists in particular, has shown great promise for the treatment of patients with PoPH, its use remains limited. As a limitation of retrospective observational studies, potential bias between groups could be inevitable. Randomized controlled trials examining the use of monotherapy and combination therapy for PoPH should be conducted to validate these findings and to take a step toward treatment standardization in patients with PoPH.

### 3.3. The role of liver transplantation in patients with PoPH

A previous review by Thomas et al. (17) emphasized the controversy surrounding the role of liver transplantation in patients with PoPH; however, recent studies have reported the beneficial effects of this treatment approach. For example, in Savale et al.’s study (46), the effects of PAH-specific therapies were examined in a large cohort of patients with PoPH from the French Pulmonary Hypertension Registry. In total, 637 patients were analyzed, 57% of whom had mild cirrhosis. PAH-specific therapy was used in 74% of patients, and survival from PoPH was significantly better in the subgroup that underwent liver transplantation. In support of these findings, Deroo et al. (53) performed a meta-analysis in which pulmonary hemodynamics and survival were examined in patients with PoPH treated with vasodilators, liver transplantation, or both. They revealed that the risk of death in patients treated with vasodilators was significantly higher than in patients who underwent vasodilator therapy combined with liver transplantation. Furthermore, in a pooled analysis of the clinical outcomes of patients from all three Mayo Clinic liver transplantation centers, 50 out of 228 patients underwent liver transplantation and showed significant hemodynamic improvement after PAH-specific therapy, with 21 patients even able to discontinue PAH-specific therapy after liver transplantation (54).

Identifying the beneficial effects of PAH-specific therapy when used in combination with liver transplantation to treat patients with PoPH is important because liver transplantation is not without its complications. For example, on reperfusion of the liver graft, pronounced systemic hemodynamic changes, such as an increase in cardiac output, are often observed (55), which can exacerbate PH and cause potential right-sided heart failure with liver graft congestion and reverse flow in the hepatic veins (56). This condition is extremely difficult to treat with existing drugs, such as milrinone, nitric oxide, and norepinephrine. For example, milrinone increases myocardial contractility, reduces systemic afterload, and reduces PVR; however, its use is limited because it can cause systemic vasodilation and resultant hypotension (57). Therefore, the use of more effective treatments preoperatively, such as macitentan or ambrisentan, which can be used alongside liver transplantation (both preoperatively and continued postoperatively as needed) provides more options to manage such patients.

Despite the controversy around the role of liver transplantation in patients with PoPH, recent studies have demonstrated clear benefits regarding survival and the ability to subsequently discontinue PAH-specific therapy. However, given the shortage of liver donors, this approach is not feasible for every patient with PoPH. Therefore, further studies are needed to identify patients with PoPH that may benefit most from liver transplantation. Jose et al. (58) suggested that PVR predicts mortality and transplantation failure in patients with PoPH; however, the exact predictors of a favorable response to liver transplantation are still unknown and should be clarified in the future.

## 4. Future perspectives

Studies examining PoPH treatment have conventionally grouped PoPH patients with idiopathic PAH patients because of the low incidence of PoPH. However, several recent studies have demonstrated differences between idiopathic PAH and PoPH in terms of hemodynamic parameters, treatment approaches, and survival, as well as socioeconomic status and healthcare utilization. Thus, grouping PoPH and idiopathic PAH may not be the best approach to pharmacotherapy studies. Instead, further multi-center trials and registry studies, such as the recent Japan Pulmonary Hypertension Registry (9), should be encouraged to ensure sufficient sample sizes to study PoPH in isolation and to obtain more specific results in this patient population. Despite both reports being conducted in Japan, Atsukawa et al. (49) reported from the hepatologist’s point of view that PAH-specific drug use is limited, while Tamura et al. (9) reported that the combination of PAH-specific drugs was useful for PoPH. Collaboration between physicians specializing in hepatology and PH may help to bridge the gap in their treatment strategies. This collaboration would drive larger studies on the use of PAH-specific therapies in patients with PoPH.

Promisingly, the potential of newer-generation endothelin receptor antagonists, such as macitentan and ambrisentan, has been demonstrated recently in patients with PoPH, without hepatic safety concerns (44, 45), which is a fundamental reason why PoPH has conventionally been excluded from clinical trials. Moreover, PAH-specific monotherapy and combination therapy have demonstrated promise by leading to significant improvements in key hemodynamic parameters, including mean PAP, PVR, and cardiac index. In the future, we hope to perform a clinical trial to examine the efficacy of endothelin receptor antagonist-based combination therapy.

In addition to PAH-specific drug therapy, liver transplantation has demonstrated beneficial effects in patients with PoPH, including improved survival and lower mortality (46, 53). Moreover, some patients were able to discontinue PAH-specific drug therapy after liver transplantation (54). However, a shortage of liver donors limits the feasibility of implementing this treatment strategy in all PoPH patients; thus, it is important to ascertain the predictors of a favorable response to liver transplantation to identify suitable candidates. It would be clinically meaningful to examine the appropriateness of early intervention for PoPH, even in patients with mild disease, as this could improve survival during and after the liver transplantation waiting period.

Regardless of the indication for liver transplantation, patients with PoPH should undergo drug therapy with PAH-specific agents. Some patients with PoPH have advanced cirrhosis, while others do not. For those that do, it would be meaningful to evaluate the impact of PAH medication on survival to liver transplantation and transplant outcomes. In patients with mild cirrhosis, treatment evaluation of PH itself should be implemented, as in other types of PAH. Furthermore, as more is learned about this disease, stratification of the background liver disease status should be examined.

## 5. Conclusion

In summary, PoPH is classified as a subtype of Group 1 PAH that is primarily seen in patients with decompensated liver cirrhosis resulting from liver disease. PoPH has a poor prognosis; however, the rarity of PoPH limits the study of this disease in isolation. PoPH is often studied with idiopathic PAH; however, recent studies suggest important differences between PoPH and idiopathic PAH. Further large-sample, multi-center trials with sufficient sample sizes are required to generate statistically meaningful results in the PoPH population. In recent clinical trials, newer-generation endothelin receptor antagonists have shown beneficial effects in the treatment of PoPH without causing hepatotoxicity. Moreover, evidence suggests that liver transplantation is beneficial in patients with PoPH, with some patients being able to discontinue PAH-specific therapy. However, the predictors of a favorable response to liver transplantation are unknown and should be examined in future studies. Collectively, future advances in these treatment strategies could help to standardize the management of patients with PoPH in the clinic.

## Author contributions

YudT, YuiT, YT, and MA wrote and edited the manuscript. All authors contributed to the article and approved the submitted version.

## Funding

This study was funded by the Japan Agency for Medical Research and Development (AMED).

## Conflict of interest

YuiT has received remuneration from Janssen Pharmaceuticals and Daiichi Sankyo, as well as research funds from Mochida. YT has received research grants from Janssen Pharmaceuticals and Nippon Shinyaku. MA has received remuneration from Janssen Pharmaceuticals.The remaining author declares that the research was conducted in the absence of any commercial or financial relationships that could be construed as a potential conflict of interest.

## Publisher’s note

All claims expressed in this article are solely those of the authors and do not necessarily represent those of their affiliated organizations, or those of the publisher, the editors and the reviewers. Any product that may be evaluated in this article, or claim that may be made by its manufacturer, is not guaranteed or endorsed by the publisher.

## Abbreviations

PAH: pulmonary arterial hypertension
PAP: pulmonary arterial pressure
PH: pulmonary hypertension
PoPH: portopulmonary hypertension
PVR: pulmonary vascular resistance
WHO: World Health Organization
